# Evaluation of Patients with Syncope in the Emergency Department: How to Adjust Pharmacological Therapy

**DOI:** 10.3390/medicina57060603

**Published:** 2021-06-11

**Authors:** Martina Rafanelli, Giuseppe Dario Testa, Giulia Rivasi, Andrea Ungar

**Affiliations:** Syncope Unit, Geriatric and Intensive Care Unit, University of Florence and Azienda Ospedaliero-Universitaria Careggi, Largo Brambilla 3, 50134 Florence, Italy; giuseppedariotesta@gmail.com (G.D.T.); giulia.rivasi@unifi.it (G.R.); aungar@unifi.it (A.U.)

**Keywords:** syncope, orthostatic hypotension, hypotensive phenotype, hypotensive susceptibility, pharmacological therapy, drugs

## Abstract

The rate of syncope in the Emergency Department ranges between 0.9 and 1.7%. Syncope is mostly related to a underlying reflex or orthostatic mechanism. A bradycardic or a hypotensive phenotype, may be identified. The latter is the most common and could be constitutional or drug induced. Consequently, obtaining an accurate drug history is an important step of the initial assessment of syncope. As anti-hypertensive medication might be responsible for orthostatic hypotension, managing hypertension in patients with syncope requires finding an ideal balance between hypotensive and cardiovascular risks. The choice of anti-hypertensive molecule as well as the therapeutic regimen and dosage, influences the risk of syncope. Not only could anti-hypertensive drugs have a hypotensive effect but opioids and psychoactive medications may also be involved in the mechanism of syncope. Proper drug management could reduce syncope recurrences and their consequences.

## 1. Introduction

Syncope is defined as a “transient loss of consciousness (TLoC) due to cerebral hypoperfusion, characterized by rapid onset, short duration, and spontaneous complete recovery” [1]. Syncope is usually classified as reflex, orthostatic or cardiac. The principal causes of syncope, which need to be addressed in the differential diagnostic process, are listed in Table 1. The prognosis varies with the type of syncope, with cardiac syncope being the most likely to lead to an increased risk of negative events [2]. Although the prognosis largely depends on the underlying cause of syncope, a syncope-related fall could be a relevant prognostic factor in all types of syncope [3,4,5,6].

Despite its frequency in the general population [2], the accurate estimation of the incidence of syncope is challenging due to the fact that different definitions have been used and because most of the patients with syncopal episodes do not seek medical assistance. However, studies conducted up to now report a frequency of syncope in Emergency Departments (ED) between 0.9 and 1.7% [7,8,9] with a hospital admission rate of up to 38% in some countries resulting in remarkable healthcare costs [10,11,12]. In addition, considering the economic burden of syncope, the readmission rate must also be taken into consideration. In one study, syncope was the most common cause of readmission, with a median cost of all-cause 30-day readmission of $26,127 [13].

In view of the above, the first medical contact, for instance in an ED, must be placed at the centre of all the strategies in order to minimize negative outcomes and to provide substantial cost savings. This proves to be particularly important when considering that the only initial evaluation may guide the diagnosis in up to 50% of the cases [14]. Indeed, the current European Society of Cardiology (ESC) guidelines on syncope [1] recommend a careful and standardized approach, which is easy to use at any age and in any clinical situation. Even if there is no independent reference standard for diagnosing, there is widespread agreement that the initial evaluation may help in distinguishing between high and low risk syncope.

Careful therapeutic recognition is the key to the initial evaluation of syncope, and should address classes of drugs, duration of treatment, relationship between drug consumption and induction of possible adverse effects. Antihypertensive drugs, diuretics, vasodilators, or pro-arrhythmic drugs can be involved in the pathophysiology of syncope [15]. This is mostly true in older adults who are usually on multiple medications.

## 2. When Should the Pharmacological Therapy Be Adjusted?

A proper risk stratification of syncope in the ED enables discrimination between discharge and admission for urgent investigation. Careful history taking, physical examination, including supine and standing blood pressure (BP) measurements, and electrocardiograms (ECG) represent the core assessment [1].

Forty to forty-five percent of non-cardiovascular and some cardiovascular life-threatening underlying conditions can be detected during ED evaluation [16]. In fact, about half of the cases of cardiac syncope are diagnosed in ED. In the other cases, a cardiac diagnosis may first be suspected and then confirmed by prolonged ECG monitoring or, less frequently, by electrophysiological study or stress test. Patients with low-risk features do not need further diagnostic tests in the ED, as they are likely to have reflex or orthostatic syncope.

Reflex/vasovagal syncope and orthostatic hypotension (OH) are the most frequent causes of transient loss of consciousness and are considered as the cardiovascular cause of orthostatic intolerance [17]. OH is defined as a fall in systolic blood pressure from a baseline value ≥20 mmHg or diastolic blood pressure ≥10 mmHg or a sustained decrease in Systolic Blood Pressure (SBP) to an absolute value <90 mmHg within 3 min of orthostatic position. When the loss of consciousness occurs on assuming the standing position, and there is concomitant significant orthostatic hypotension, orthostatic syncope is confirmed [1]. 

A bradycardic and a hypotensive phenotype of syncope may also be identified [18].

A “low blood pressure phenotype” identifies patients in whom a chronic low BP plays a role in causing orthostatic reflex syncope and orthostatic intolerance [1]. The hypotensive phenotype is the most common mechanism of syncope and could be constitutional [19,20] or drug-related, when BP is constantly below the target range in patients on antihypertensive treatment. 

Hypotensive drugs may exacerbate hypotensive susceptibility, especially as vulnerability to vertical posture stress [21]. In addition, orthostatic vasovagal syncope, situational reflex syncope, vaso-depressive and mixed carotid sinus syndrome, OH-related syncope, postprandial hypotension, reflex syncope triggered by tachyarrhythmia and recurrent severe hypotensive episodes during ambulatory BP monitoring (daytime SBP <90 mmHg), may all be related to hypotensive susceptibility [1].

Patients prone to reflex syncope may thus be more sensitive to antihypertensive medication, resulting in a greater BP drop. Moreover, orthostatic hypotension is most commonly drug-related, particularly in older hypertensive patients on three or more hypotensive medications [22,23,24], in whom a systolic BP <100 mm Hg is considered inappropriately low [25,26].

The bradycardic phenotype is likely when syncope occurs during a spontaneous or induced asystole lasting more than 3 s [27]. However, syncope due to cardiac arrhythmias or to structural heart disease, syncope occurring in the supine position, vasovagal syncope triggered by emotional distress (blood phobia, instrumentation, visceral or somatic pain), or cardio-inhibitory reflex syncope are less likely to be related to hypotensive medications.

In view of the above, drug adjustment is a crucial step in the evaluation of patients with hypotensive syncope.

## 3. How to Adjust Medication?

Vaso-active and cardio-active drugs may hinder the compensatory reflex responses to standing (e.g., sympathetic-mediated vasoconstriction and increased heart rate response), increase venous pooling (e.g., vasodilators) and/or induce volume depletion (e.g., diuretics), thus favouring OH, which can trigger a reflex bradycardia, when delayed.

In hypertensive patients with drug-related hypotension, the physician should tailor the pharmacological therapy so as to balance between the risk of cardiovascular events and the risk of syncope recurrence.

In patients with recurrent and severe episodes of syncope, especially when older and frail, blood pressure lowering medication should be prescribed with caution. Previous studies have demonstrated a decrease in syncopal recurrences after the reduction or discontinuation of anti-hypertensive medications in adults and older patients, with no safety concerns [28,29,30,31]. Thus, tailoring prescriptions of blood pressure drugs to age, frailty, disability, risk of cardiovascular events and syncope relapses has been proposed (Table 2).

Not only could anti-hypertensive drugs have a hypotensive effect, but also drugs that might increase hypotensive susceptibility, as listed in Table 3.

## 4. Vaso-Active and Cardio-Active Drugs

**Figure 1:**
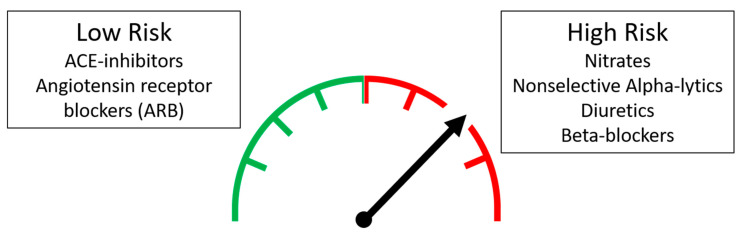
Antihypertensive medications that may increase the risk of orthostatic hypotension.

Anti-hypertensive medications are known predisposing factors for OH, through the above-mentioned mechanisms. However, in the light of the available scientific evidence, some “higher risk” classes of drugs have been identified (Figure 1):

Nitrates. Nitrates stimulate nitric oxide release, inducing smooth muscle vascular wall relaxation, venous vasodilation, and therefore reduction in preload. Nitrates significantly increase the risk of orthostatic syncope, regardless of dosage and comorbidities [33,34,35,36].

Nonselective alpha-receptor adrenergic antagonists (or alpha-lytic). Alpha-receptor adrenergic antagonists inhibit the vasoconstrictor effect mediated by catecholamines through the selective blockade of α1-adrenergic receptors and thus induce arterial and venous vasodilation, with the consequent reduction of peripheral resistance and increased venous capacitance (mediated by binding to 1B receptors). 

Alpha-lytics facilitate bladder emptying by binding 1A receptors in the prostate and bladder, and thus are widely used in the treatment of urinary tract obstruction. Numerous studies conducted in different clinical settings have demonstrated the role of alpha-lytics as determinants of OH in the elderly [37,38,39]. Alpha1-receptor blockage counteracts the vasoconstriction that occurs upon standing [40]. Highly selective molecules such as silodosine and tamsulosine reduce the risk of hypotension, which is greater for less selective molecules such as alfuzosin, terazosin and doxazosin [41].

Diuretics. Diuretic therapy can increase the risk of orthostatic hypotension by inducing volume depletion, which may be sufficient to alter the BP response to standing, especially in the elderly [35]. In addition, loop diuretics have been significantly associated with orthostatic hypotension in patients with syncope [42].

Beta-blockers. Beta-blockers interfere with BP response when standing, counteracting heart rate, cardiac output increase, and sympatho-mediated vasoconstriction upon standing. Therapy with beta-blockers was associated with an increased risk of initial orthostatic hypotension [43].

“Low risk” classes of drugs can be identified as the following:

ACE-inhibitors and angiotensin II receptor blockers (ARB). ACE-inhibitors reduce angiotensin II and increase kinin levels, both inducing vasodilation. 

ARBs are competitive antagonists of angiotensin II, type 1 receptors. Studies that investigated the effect of these two classes of drugs on BP pressure response upon standing have not identified a close association with orthostatic hypotension [43,44,45]. On the contrary, some data seem to suggest a protective effect of both ACE-inhibitors [44,46] and ARBs [47].

Areas of Uncertainty:

Calcium channel blockers. Data are conflicting. A protective effect of dihydropyridine calcium channel blockers on OH has been reported [48], while other studies suggest increased risk factors [47,49]. Non-dihydropyridine calcium channel blockers have negative chronotropic and inotropic effects, which may impede heart rate compensatory responses to standing [23].

Clonidine. Little is known about the impact of clonidine on BP response upon standing. It is not clear whether clonidine is a protective or a risk element for OH [50].

Other Medications

In addition to cardiovascular medications, other drugs may have a hypotensive effect and may interfere with reflex responses to standing. 

Orthostatic hypotension is a potential side effect of tricyclic antidepressants, trazodone and antipsychotics due to alpha-adrenergic receptor blockade [47,49,51]. 

Selective serotonin reuptake inhibitors and benzodiazepines have been associated with abnormal upright BP responses [52,53]. 

Antiparkinsonian drugs such as levodopa and selegiline can lead to OH, inducing vasodilation and reduced sympathetic outflow [54,55]. 

Opioids can induce histamine release, causing vasodilation and increased capillary permeability, resulting in BP reduction [51,56]. Opioid administration may be associated with OH and syncope and it is mainly prescribed as buprenorphine, methadone, oxycodone and tapentadol [56].

## 5. Specifically, What Procedures Should Be Carried Out in the ED?

According to ESC guidelines of syncope [1], the ED physician must first rule out arrhythmic or structural cardiac causes of syncope. 

After having excluded cardiac syncope, the physician should define whether the syncope is likely OH-related or neurally-mediated. In both cases, patients need to be instructed on appropriate hydration, physical countermeasures, physical conditioning and the use of compressive stockings in OH. This is the core management.

In the case of drug-related hypotension and/or orthostatic hypotension, it is reasonable to consider the withdrawal or reduction of hypotensive medications after evaluating cardiovascular risks. Considering changing molecules or therapy regimen should be an alternative when it is not possible to withdraw a medication. 

Practical details are shown in Table 4

## 6. Conclusions

Reflex syncope and orthostatic hypotension are the most frequent causes of transient loss of consciousness, considered as a cardiovascular cause of orthostatic intolerance. 

Anti-hypertensive, psychoactive medications, opioids and other classes of drugs have vaso-active effects and might predispose a patient to orthostatic hypotension and syncope. 

Accurate therapeutic recognition is an important step in the assessment of syncope. Proper management of the pharmacological therapy could reduce syncope recurrences and their consequences.

## Figures and Tables

**Table 1:** Causes of syncope, adapted from Moya A. et al. [1].

**Reflex syncope**
Vasovagal (VVS) orthostatic VVS: standing, or less common sitting emotional: fear, pain, instrumentation, blood phobia pain triggers: peripheral or visceral
Situational micturition gastrointestinal stimulation cough, sneeze others (e.g., laughing, brass instrument playing, weight lifting, post-exercise)
Carotid sinus syncope
**Orthostatic Syncope**
Drug-induced orthostatic hypotension
Volume depletion Primary autonomic failure (pure autonomic failure, multiple system atrophy, Parkinson’s disease, dementia with Lewy bodies)
Secondary autonomic failure (diabetes, amyloidosis, spinal cord injuries, auto-immune autonomic neuropathy, paraneoplastic autonomic neuropathy, kidney failure)
**Cardiac syncope**
Arrhythmia as primary cause: Bradycardia: - sinus node dysfunction - atrioventricular conduction system disease - implanted device malfunction Tachycardia: - supraventricular - ventricular
Structural disease: cardiac valvular disease, acute myocardial infarction/ischaemia, hypertrophic cardiomyopathy, cardiac masses (atrial myxoma) pericardial disease/tamponade, congenital anomalies of coronary arteries, prosthetic valves dysfunction.
**Cardiopulmonary and great vessels**
Pulmonary embolus, acute aortic dissection, pulmonary hypertension

**Table 2:** BP targets in patients with hypertension and treatment-related syncope, adapted from Rivasi et al. [31].

	Age < 70	Age > 70 or Frailty	Disability
Low syncope risk and high CV risk	120–130 mmHg	130–140 mmHg	<160 mmHg
High syncope risk and low CV risk	130–140 mmHg		

**Table 3:** Vaso-active drugs, adapted from Gibbons C.H. et al., 2017 [32].

Classes of Drugs	Drugs
Dopaminergic agents	levodopa, dopamine agonists
Antidepressant	amytriptiline, nortryptiline, imipramine, desipramine
Anticholinergics	atropine, glycopyrrolate, hyoscyamine
*Antihypertensives*	
Diuretics	furosemide, torsemide, acetazolamide, spironolactone, hydrochlorothiazide
Nitrates	nitroprusside, isosorbide dinitrate, nitroglycerin
Phosphodiesterase E5 inhibitors	sildenafil, vardenafil, tadalafil
Alpha-1 adrenergic antagonists	alfuzosin, doxazosin, tamsulosin
Dihydropyridine calcium channel blockers	amlodipine, nifedipine, nicardipin
Other direct vasodilators	hydralazine, medoxidil
*Negative inotropic/chronotropic agents*	
Beta-adrenergic blockers	metroprolol, propranolo, atenolol, bisoprolol, etc.
Non dihydropyridine calcium channel blockers	verapamil, diltiazem
*Central sympatolytic agents*	
Centrally acting alpha-2 agonists	clonidine
False neurotransmitters	alpha-methyldopa
*Renine-angiotensin system (RAS) antagonists*	
ACE inhibitors	captopril, enalapril, perindopril
ARB	losartan, telmisartan, candesartan

**Table 4:** Key points for the management of hypotensive medications in syncope.

Key Points
1. Considering the reduction or withdrawal of hypotensive medication.
2. Considering changing molecules or therapy regimen (preferring bedtime administration, except for diuretics) when it is not possible to withdraw a hypotensive medication.
3. Preferring selective beta-blockers instead of alpha- and beta-receptor blockers, when indicated.
4. Preferring uro-selective alpha-lytics in patients with BPH-associated LUTS (e.g., silodosin), when indicated.
5. Avoiding diuretics, unless specifically indicated as essential.
6. Considering renal and hepatic impairment in order to avoid drug accumulation.

BPH: benign prostatic hyperplasia; LUTS: low urinary tract symptoms.
